# LUHMES Cells: *In Vitro* Model for Studying Tauopathy in Alzheimer’s Disease

**DOI:** 10.1002/alz.091874

**Published:** 2025-01-03

**Authors:** Madeline Moffett, Mayur S Parmar

**Affiliations:** ^1^ Nova Southeastern University Dr. Kiran C. Patel College of Osteopathic Medicine, Clearwater, FL USA; ^2^ Nova Southeastern University Dr. Kiran C Patel College of Osteopathic Medicine, Clearwater, FL USA

## Abstract

**Background:**

Alzheimer’s disease (AD) is a common neurodegenerative disorder that results in the accumulation of amyloid‐beta, neurofibrillary tangles, and progressive cognitive decline. Despite extensive research into the pathophysiology of AD and potential treatments, a definitive cure remains elusive. Appropriate *in vitro* cell models are crucial for understanding pathophysiology and drug screening for AD. Cell lines like HEK293, HeLa, SH‐SY5Y, and PC have been used to study AD pathology. However, these models lack neuronal cell characteristics and do not express endogenous tau, a microtubule‐associated protein implicated in AD pathophysiology. The Lund Human Mesencephalic (LUHMES) cell line is a promising *in vitro* neuronal cell model that expresses endogenous tau protein, allowing a deeper exploration of tau pathophysiology and the assessment of biotherapeutics.

**Method:**

The data was collected through the PubMed database. Review articles were selected based on the following keywords: “*in vitro* tauopathy,” “LUHMES and neurodegenerative disease,” “LUHMES and tauopathy,” “LUHMES and Alzheimer’s disease,” and “neuron cell line.” Articles utilized for the purpose of this study were primary research articles published in academic journals between 2014 and 2023.

**Result:**

The LUHMES cell line is used to model known tauopathies, like AD and progressive supranuclear palsy (PSP), yet few studies have harnessed their potential. One study revealed that PP242, a dual inhibitor of mTORC1 and mTORC2, could reverse fenazaquin‐induced tau aggregation in LUHMES cells. Another study discovered that activating PERK, an endoplasmic reticulum stress sensor, reduced tau phosphorylation and aggregation in LUHMES cells. As human‐origin cells with neuronal properties, LUHMES cells provide an essential model for studying tau pathology in AD. The insights gathered from these LUHMES‐based studies highlight the utility and relevance of these cells in propelling our understanding of tauopathy forward.

**Conclusion:**

The LUHMES are an important neuronal cell line that can be utilized for *in vitro* experiments to study tauopathy and evaluate biotherapeutics for their ability to modulate tau pathology. These cells have endogenous tau expression, a key characteristic differentiating this line from more commonly used cell lines, such as HEK293 and SH‐SY5Y. Significant progress toward evaluating effective treatments can be made by using the LUHMES cell line to study AD‐related tauopathy pathology.

